# Multilevel Breast Reduction: A Retrospective Study of 338 Breast Reduction Surgeries

**DOI:** 10.1097/GOX.0000000000002427

**Published:** 2019-08-30

**Authors:** Amiram Borenstein, Or Friedman

**Affiliations:** From the * Borenstein Plastic Surgery Clinic, Tel-Aviv, Israel; † Sackler Faculty of Medicine, Tel-Aviv University, Tel-Aviv, Israel.

## Abstract

Aesthetic breast reduction is a common plastic surgery procedure with the potential for considerable improvement of the patient’s quality of life in addition to its aesthetic value. Many different approaches have been described for breast reduction to improve upon previous techniques in terms of scarring, nipple position, amount of breast tissue excised, shape, and longevity of the results. However, medium size resections in ptotic and wide breasts are difficult to treat using both the most common inferior pedicle wise procedure and the various vertical scar techniques.

**Methods:**

A retrospective study of 338 consecutive bilateral breast reduction patients between January of 2010 and January of 2018 at a single center by a single surgeon using a vertical scar technique. Demographic and postoperative outcome data were collected and evaluated.

**Results:**

Patient satisfaction with the results was high. Complication rates were comparable or lower than previously published series. Major complications requiring revision surgery: 3 (0.8%) hematomas; minor complications: 68 (20%) cases of superficial dehiscence; 12 (3.5%) superficial surgical site infections; 11 (3.2%) seromas; 4 (1.2%) fat necrosis; and 1 (0.2%) partial areola necrosis.

**Conclusion:**

The Borenstein Breast Reduction technique aims to recreate the breast mound support from the “bottom up” facilitating long-lasting results and high patient satisfaction rates. This approach can be helpful in all breast reductions and is most effective in wide ptotic breasts.

According to the ASPS national plastic surgery statistics, 43,591 breast reduction procedures were performed during 2018.^1^ Patients seeking breast reduction come in all shapes and sizes. Common medical complaints include back pain, IMF fungal infection, intertrigo, irritation, and even abrasion wounds from the bra suspenders on the shoulders.^2–5^ Most patients claim they do not like their breast. Some patients avoid swimwear, have difficulties shopping for bras, or merely wish they could wear a buttoned blouse.^2–5^

Several landmark evolutions in breast reduction influence how most surgeons perform these procedures. The year 1956 marked the era of modern breast reduction when Wise^2^ described a skin resection pattern adapted from a brassiere design, which became known as the inverted T. Pitanguy^3^ introduced the superior pedicle during the 1960s. The vertical scar technique for reduction mammaplasty was first described in 1925 but popularized by Lassus^4^ and soon after modified by Lejour^5^ limiting the scar to a vertical pattern. This approach also enhanced breast shape by reducing the boxy appearance that sometimes occurred with the inverted T. The vertical skin incision pattern has been adapted to inferior,^6^ medial,^7^ superomedial,^8,9^ and lateral pedicles.^10,11^

However, despite the plethora of modifications and innovations, both the wise pattern and the vertical scar techniques fall short when applied to medium resections in ptotic wide breasts.

The purpose of this study was to examine the clinical outcomes, complication, and revision rates among a single surgeon’s consecutive cases.

## MATERIALS AND METHODS

A retrospective study of 338 consecutive bilateral breast reduction patients between January of 2010 and January of 2018 from the private clinic of the first author (A.B.). Demographic and postoperative outcome data were collected and evaluated. (Video, which displays the Borenstein Breast Reduction technique.)

### 

Video.

The breast is marked as a vertical scar reduction.

After marking, preparing, and draping the supine patient de-epithelization of a slightly smaller area then the premarked new areola is done. Next, the central wedge resection skin incision is made with a scalpel followed by electrocautery dissection down to the pectoralis fascia. Next, undermining of the breast tissue above the pectoralis fascia is extended, as would be done for a subglandular implant pocket. One 3 absorbable Vicril 0 (Johnson & Johnson Medical N.V., Diegem, Belgium) sutures are put between the lateral breast tissue and pectoralis fascia, just medial to the breast meridian at 30 degrees upward angle. Important, the former sutures cannot be tied down before its effect on the lateral breast slope. The previous step can be repeated more superficially with^2,3^ sutures in larger breasts. One 2 Pillar sutures are added to approximate the pillars loosely.

Next, the Borenstein maneuver is performed; 2 thin dermal flaps, similar to facelift skin flaps, are developed on either side above the pillars. Two 4 horizontal figure of 8 sutures are put at the freshly exposed breast tissue edges above the pillars, narrowing the breast while adding projection. The previous step is repeated as needed. When the wanted breast shape is achieved, the excess thin skin strips are cut, and the tension-free dermal edges are approximated using an absorbable intradermal suture. The patient is then seated, and an external suture is put to mark the bottom of the future areola. A dyed cookie cutter is placed over the nipple-areola complex and surrounding skin to mark the new areola opening. De-epithelization of the areola opening is made, and insetting of the nipple-areola complex is completed.

## RESULTS

Three hundred thirty-eight consecutive patients underwent bilateral breast reduction performed by a single surgeon during the study period. The mean age was 34 years (range: 18–66 years; Table 1). Major complications requiring revision surgery: 3 (0.8%) hematomas; minor complications treated by local antibiotic ointment application: 68 (20%) cases of superficial dehiscence^12^; (3.5%) superficial surgical site infections^11^; (3.2%) seromas^4^; (1.2%) fat necrosis^1^; and (0.2%) partial areola necrosis.

**Table 1.** Demographic and Clinical Data of Patients

Patients Demographic Data (n = 338)	
Average age	34
Had previous pregnancies	146
Smokers	51
Average resected tissue weight—right, g	395
Average resected tissue weight—left, g	400
Average SN to nipple distance—right, cm	29
Average SN to nipple distance—left, cm	29
Average IMF to nipple distance—right, cm	13
Average IMF to nipple distance—left, cm	13

SN, sternal-notch nipple; IMF, infra-mammary fold.

## DISCUSSION

According to the American Society of Plastic Surgeons national plastic surgery statistics, 43,591 breast reduction procedures were performed during 2018.^1^

As with any procedure that does not have 1 ideal method, the history of breast reduction is replete with different procedures—all of which have limitations.^2–10^

Most of the cited methods debulk the breast tissue, taking care to preserve the nipple-areola complex, and pack it all in with a tailored skin “brassiere.” Some authors have attempted to address the shaping of the central portion of the breast via pillar suture or suspending the pedicle in attempts to augment the upper pole. However, essentially the “construction” of the reduced breast could be likened to building a house from the roof down, sometimes without even addressing its foundations.

**Fig. 1.**
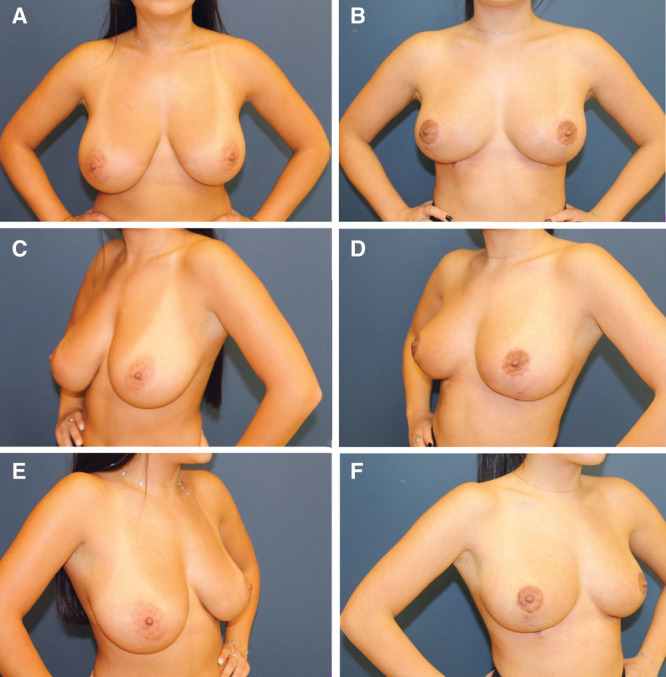
Before and after photos of an 18.5-year-old patient at 6-month follow-up. A, Before, hands-on waist anterior posterior (AP). Asymmetric low hanging and laterally displaced breasts. B, Six-month follow-up, hands-on waist AP. Demonstrating a better width to length ratio. C, Before, hands-on waist lateral. Flattening of the lateral breast curve into the axilla. D, Six-month follow-up, hands-on waist lateral. Creation of the lateral breast curve with an almost implant-like projection. E, Before, hands-on waist lateral. Flattening of the lateral breast curve into the axilla. F, Six-month follow-up, hands-on waist lateral. Only on this side, the vertical scar which lengthened onto the chest wall is readily apparent.

The Borenstein Breast Reduction (BB-R) technique aims to shape the breast mound from the “bottom up” (Fig. 1). The technique follows the rational markings of a vertical scar breast reduction. Initial skin and breast tissue excision are limited to the vertical limbs set by the “Lasuss maneuver.” The breast mound foundation is set by lateral to medial absorbable sutures, bringing in the lateral breast tissue and fixing it to the pectoralis fascia. Building upon the new breast mound foundation, the “Borenstein maneuver” is applied in a stepwise manner to incrementally expose superficial breast tissue and snugly invaginate it over the approximated pillars. This step is critical, in that it translates the horizontal breast access to projection. Moreover, the stepwise execution of the maneuver facilitates an intuitive shaping of the breast, similar to a child building a sand castle on the beach. The figure of 8 sutures aids in the vertical arrangement of the collected tissue. When the wanted projection is achieved, the skin flaps raised above the now invaginated breast tissue are excised to facilitate tension-free closure, and at this point, the bottom of the areola is marked with a suture. The final placement of the areola, reminiscent of original description by Pitanguy,^3^ helps in setting what will be the focal point of the breast in best-controlled manner, per the aesthetic relations of the breast.^14^ The final result targets 3 previously undertreated characteristics of most hypertrophic breasts: (1) lateral displacement of the breast; (2) correction of wide breast base; and (3) projection.

In our series, complication rates are comparable or lower than previously published series.^2–11^ The relatively high rate of superficial dehiscence (68 patients, 20%) could be explained by our strict reporting criteria, which included every patient who was prescribed any local treatment following follow-up visits. Typically, the areas of superficial dehiscence were located at the junction between the bottom part of the areola and the vertical scar. Their size ranged from a pinpoint opening marked by a delicate crust to a 3 mm opening in the scar with part of the intradermal Monocryl suture knot showing. None of our cases needed any form of reclosure in office or otherwise. The 3 (0.8%) patients taken back to revision surgery in our series suffered from hematomas. We do not routinely use drains or liposuction in breast reduction surgery and are highly suspicious of any “bruising” or irregular swelling after surgery.

**Fig. 2.**
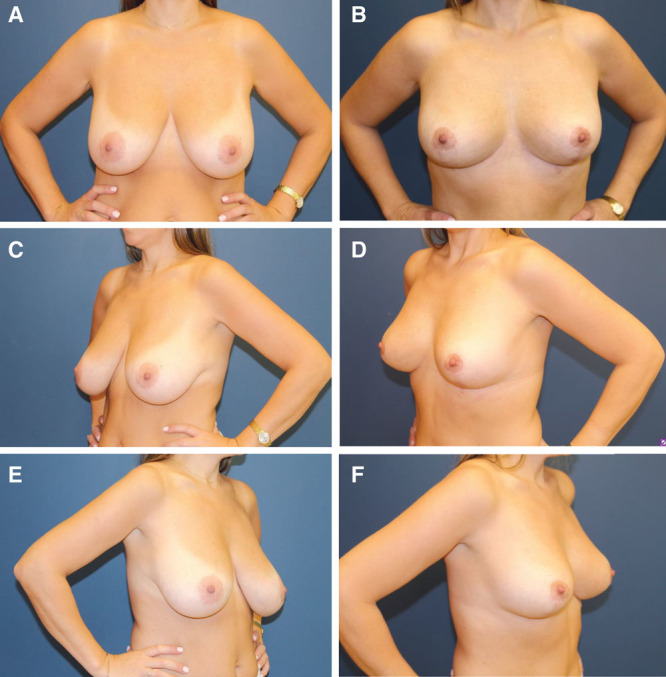
Before and after photos of a 37-year-old patient at 6-month follow-up. A, Before, hands-on waist AP. Low hanging breasts, the empty upper pole, and how the breast seems flattened against the chest wall. B, Six-month follow-up, hands-on waist AP. Better width to length ratio. C, Before, hands-on waist lateral. Flattening of the lateral breast curve into the axilla. D, Six-month follow-up, hands-on waist lateral. Lateral breast curve with an almost implant-like projection. Also, the vertical scar which lengthened onto the chest can be seen on this view. E, Before, hands-on waist lateral. Flattening of the lateral breast curve into the axilla. F, Six-month follow-up, hands-on waist lateral.

**Fig. 3.**
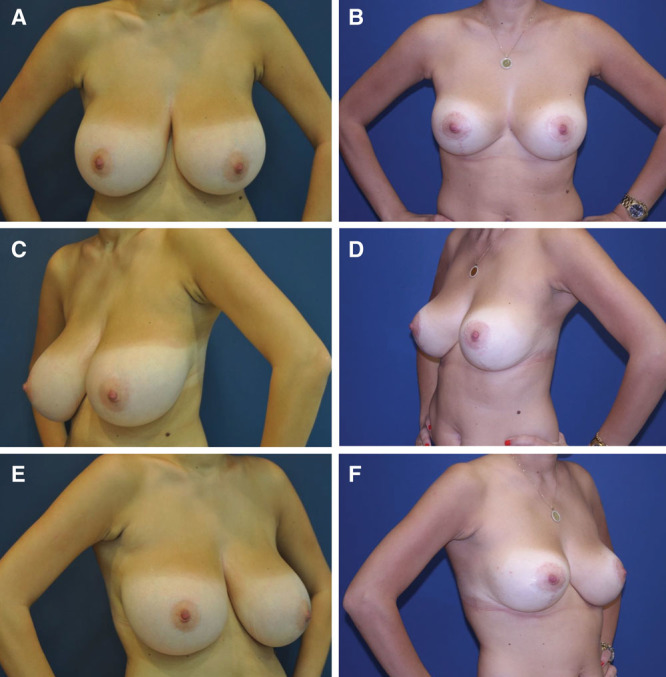
Before and after photos of a 21-year-old patient at 5-year follow-up. A, Before, hands-on waist AP. Wide heavy breasts, nipples-areola complex considerably low under the inframammary fold. B, Five-year follow-up, hands-on waist AP. Considerable reduction in width while maintaining a pleasing lateral slope. C, Before, hands-on waist lateral. Wide breast is continuing into the axilla. D, Five-year follow-up, hands-on waist lateral. Significant narrowing of the breast and creation of the lateral breast curve with an almost implant-like projection preserved for 5 years in an active, outgoing woman. E, Before, hands-on waist lateral. Flattening of the lateral breast curve into the axilla. F, Six-month follow-up, hands-on waist lateral. Mature vertical scar can hardly be noticed beyond the IMF on any of the views.

This technique may be applied to a wide variety of patients, as can be seen by the varied selection of patients (Table 2). However, its real strength is evident when applied to patients with medium-sized ptotic (both inferiorly when standing and laterally when laying on the back) and wide breasts (Figs. 1–3) in which any other attempt would either fail to create projection or substantially increase the complication rates.

**Table 2.** Summary of the Complications

Complication	n = 99 (%)
Minor dehiscence	68 (20)
Infection	12 (3.5)
Seroma	11 (3.2)
Fat necrosis	4 (1.2)
Hematoma	3 (0.8)
Partial areola necrosis	1 (0.2)

## TIPS AND TRICKS TO THE TECHNIQUE

1. Markings and de-epithelization are similar to any superior breast lift (Video, which displays Borenstein Breast Reduction technique).
2. Following the central wedge resection, undermining of the breast tissue above the pectoralis fascia enables the reorganization of the breast mound foundation, and it helps to imagine the implant the surgeon would have picked for the patient considering her chest width and habitus.
3. Medialization of the laterally displaced breast tissue using Borenstein sutures controls the lateral vector of breast ptosis and creates the foundation for a pleasing new lateral slope and narrow base for the rest of the breast.
4. Layered closure of the vertical scar converts breast width to projection, aids final NAC positioning, and reduces tension from final intradermal skin sutures.
5. NAC opening final de-epithelization and placement is done as the last step when the breast mound is reconstructed, and its position can be verified in a 3D manner.

There are several limitations associated with the BB-R technique. First, the lateral sutures are essential for building the foundation of the breast mound; these sutures may lead to dimpling in the lateral breast. These are difficult to treat if not identified during placement, and often a compromise on breast shape should be made if they are to be released. Second, the vertical scar may, at times, lengthen to the abdominal wall. We appreciate the logic behind the approach by Hall-Findlay and Shestak^14^ and use of liposuction as an adjunct to breast reduction procedures. We do not routinely perform liposuction during a breast reduction, so we cannot remark on its merits, perhaps this may help in the final shaping of the lower pole of the breast and facilitate further skin tightening in selected patients. In patients with poor skin quality conversion to limited inverted T scar may be prudent. In our experience, scars extending beyond the IMF to the superior abdominal wall are well tolerated by our patients, as long as they have been appropriately informed, and the scar is hidden by the inferior pole of the breast when their hands rest alongside their body.

## CONCLUSIONS

The BB-R technique is a safe, reproducible surgical option which addresses some of the previously undertreated problems of oversized breast—wide, laterally displaced breast tissue. Building the breast mound from the “bottom up” enables the surgeon to form the breast and treat the tissue dynamically. This is a powerful technique to add to the breast surgeon’s surgical toolbox.
